# Evaluation of antimicrobial photodynamic therapy with acidic methylene blue for the treatment of experimental periodontitis

**DOI:** 10.1371/journal.pone.0263103

**Published:** 2022-02-10

**Authors:** Juliano Milanezi de Almeida, Henrique Rinaldi Matheus, Breno Edson Sendão Alves, David Jonathan Rodrigues Gusman, Maria José Hitomi Nagata, Elisa Mara de Abreu Furquim, Edilson Ervolino

**Affiliations:** 1 Periodontics Division, Department of Diagnosis and Surgery, School of Dentistry, São Paulo State University (Unesp), Araçatuba, Brazil; 2 School of Dentistry, Nucleus of Study and Research in Periodontics and Implantology (NEPPI), São Paulo State University (Unesp), Araçatuba, SP, Brazil; 3 Department of Basic Science, School of Dentistry, São Paulo State University (Unesp), Araçatuba, Brazil; University of Life Sciences in Lublin, POLAND

## Abstract

**Objective:**

To investigate the security and effectiveness of antimicrobial photodynamic therapy (aPDT) with a citric acid-based methylene blue (MB) on the periodontal repair following the treatment of ligature-induced experimental periodontitis (EP) in rats.

**Material and methods:**

Were used 120 male rats, randomly divided into 4 experimental groups (n = 30): no treatment (NT), SRP alone (SRP), SRP plus aPDT using conventional MB pH 7.0 (aPDT-pH7), SRP plus aPDT using acidic MB pH 1.0 (aPDT-pH1). EP was induced at day 0 by the placement of a ligature around the mandibular left first molars. Ten animals per group/period were euthanized at 14, 22 and 37 days. Histopathological, histometric (percentage of bone in the furcation [PBF]) and immunohistochemical (for tartrate-resistant acid phosphatase [TRAP] and osteocalcin [OCN]) analyses were performed. Data were statistically analyzed.

**Results:**

aPDT-pH1 showed the highest PBF as compared with the other treatments. Collectively, tissues’ reaction to both dyes were controlled and healthy for the periodontium. Both aPDT protocols reduced the extent and intensity of the local inflammatory response, reduced the alveolar bone resorption, and promoted a better structural arrangement of the connective tissue as compared with SRP. TRAP expression was downregulated while OCN expression was upregulated by aPDT as compared with SRP alone.

**Conclusion:**

Our data implicate that the novel MB pH 1.0 is as safe as the conventional MB for use in aPDT and raises its additional benefit of increasing the amount of alveolar bone in the furcation.

## Introduction

Periodontitis is a highly prevalent oral disease in the United States, estimated to affect over 42% of dentate US adults 30 years or older [1], and the primary cause for tooth loss in the world [2]. Periodontal breakdown is the consequence of the combination of complex illnesses, interactive amongst inflammatory and immunologic systems of a host, subgingival microbiota, and environmental factors [3]. Even disorders within this microbiota being capable to result in dysbiosis, a specific microenvironment and susceptibility are essential to trigger the disease [4]. Hence, periodontal therapy will be the most effective when capable to encompass the many variables as possible.

The use of antimicrobial photodynamic therapy (aPDT) has been increasingly investigated as an adjunctive approach to non-surgical scaling and root planing (SRP) [5] for the treatment of periodontitis in sites with impaired access, such as deep periodontal pockets and furcation areas [6, 7]. The impact of photobiomodulation therapy ranges from biomolecules (e.g. nucleic acids, amino acids, carbohydrates) to entire organisms [8]. In aPDT, the reactive oxygen species released from the interaction between light at a specific wavelength and photosensitizer (PS) cause irreversible damage to the cytoplasmic membrane and DNA of microorganisms [9, 10].

Despite the benefits of aPDT [8], the positive effects found in animal experiments do not represent potential clinical relevance in human studies [11]. The complexity of periodontitis, limited reachability to biofilms, and the focus of aPDT in one variable (i.e. microbiota) lead to this scenario, and, hence, the proposition of improvements in aPDT may increase the clinical relevance of the therapy. Therefore, aiming to improve the outcomes obtained with aPDT, the literature reports alterations to its initial protocol, such as multiple aPDT sessions [12], distinct light sources [13, 14] and PS [15], and incorporation of nanoparticles [16].

The rate of growth, cell wall composition, and presence of polysaccharide intercellular adhesin (PIA) differ between cells growing in biofilms and their planktonic forms, which may block the penetration of the photosensitizer and light to the deeper layers and thereby reduce the photosensitizing process [17]. This way, alterations in PS in order to target this topic may enhance its antimicrobial properties. Ruggeri Jr et al. [18] supported the hypothesis that manual or ultrasonic instrumentation alone is not able to expose the sound dentin matrix, whereas a subsequent citric acid conditioning exposes collagen fibrils and associated proteoglycans, indicating deeper penetration into mineralized tissues. Also, in addition to the reduced oral biofilm formation achieved with citric acid etching [19], root conditioning with citric acid promotes the adhesion of the fibrin network onto the dentin surface, which may further enhance connective tissue attachment to the root surface [20].

Animal experimentation is known to be the first step for *in vivo* validation [21]. In periodontics, rodent models of experimental periodontitis allow not only the understanding of the pathogenesis [22] but also the validation of hypotheses related to therapies for periodontal treatment [7].

Substantiated by the favorable properties of citric acid on mineralized tissues and reduction of oral microorganisms formation (both related with periodontal treatment and repair), and based on the increased biocompatibility of methylene blue (MB) acidulated with citric acid pH 1.0 over conventional MB (pH 7.0) [23], this *in vivo* experiment aimed to assess the hypothesis that a novel acidic MB could provide additional benefits over conventional MB, used in aPDT as adjunctive therapy to SRP for the treatment of experimental periodontitis (EP).

## Materials and methods

### Animals

**Fig 1:**
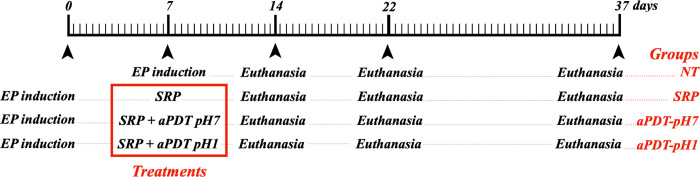
Scheme illustrating the experimental procedures performed during the study for each group.

The study followed a randomized, single-blind, controlled design, and was conducted in accordance with the ARRIVE Guidelines: Animal Research: Reporting In Vivo Experiments [23]. The protocols were approved by the Ethics Committee on Animal Use under protocol the 310–2016 of São Paulo State University, UNESP, School of Dentistry, Araçatuba. Previous experience of our research group [7] determined n = 10 enough to reject the null hypothesis in the histopathological, histometric, and immunohistochemical analyses. One hundred twenty healthy 3‐month‐old male rats (*Rattus norvegicus*, *albinus*; Wistar) weighing 250–300 g were kept in plastic boxes with wood shavings, under 12 hr/12 hr light/dark cycles, 22 ± 2°C ambient temperature, 20 air changes per hour, 55 ± 5% humidity, receiving feed and water ad libitum, and monitored daily. A blinded staff external to the study used the Minitab® 17 software (Minitab Inc., State College, PA, USA) to perform simple randomization (1:1 allocation ratio) of the animals to one of the four experimental groups: no treatment (NT), SRP, aPDT-pH7 and aPDT-pH1 (Fig 1). The euthanasia were performed at 14, 22, or 37 days after EP induction in groups SRP, aPDT-pH7, and aPDT-pH1, with an overdose (150 mg/kg) of sodium thiopental (Cristália Ltda., Itapira, SP, Brazil), consisting of ten animals per period.

- Group NT (n = 30): EP induction and no treatment performed (ligatures remained around the mandibular left first molars during the entire experiment);
- Group SRP (n = 30): EP induction and treatment with SRP and subgingival irrigation with physiological saline solution (PSS), 7 days after EP induction;
- Group aPDT-pH7 (n = 30): EP induction and treatment with SRP plus aPDT using conventional MB pH 7.0 (100μg/ml)), 7 days after EP induction;
- Group aPDT-pH1 (n = 30) EP induction and treatment with SRP plus aPDT using a novel citric acid-based MB pH 1.0 (100μg/ml), 7 days after EP induction.

### Anaesthesia

For all experimental procedures, the animals were anesthetized via intramuscular injection with xylazine hydrochloride (6 mg/kg of body weight) and ketamine hydrochloride (70 mg/kg of body weight).

### Experimental periodontitis induction

At day 0, a #24 cotton thread (Cotton Chain N°. 24, Coats Corrente, São Paulo, SP, Brazil) was placed around the mandibular left first molars to induce EP [7, 24] in groups SRP, aPDT-pH7, and aPDT-pH1. In group NT, EP was induced at day 7 by using the same method.

### Preparation of the dyes

The conventional PS was prepared (MB 100 μg/ml) in distilled water at native (not adjusted) pH 7.0. Initially, for the novel PS, was prepared an unsaturated solution of citric acid adjusted to pH 1.0. After preparation, this solution was used as a solvent for the MB in order to achieve a concentration of 100 μg/ml. At the moment of the experiment, the pH of the dye was rechecked and kept stable.

### Treatments

Were performed by the same calibrated operator [JMA], masked to the experimental groups, 7 days after EP induction. SRP was conducted in accordance with the protocol described by Almeida et al. (2008) [7]. In groups treated with aPDT, either the MB pH 7.0 or pH 1.0 were carefully inserted and kept within the periodontal pocket for 60 s and then irradiated with a red (AlGaInP) diode laser (TheraLase—DMC, Sao Carlos, SP, Brazil) in one point at the buccal and one point at the lingual surfaces of the mandibular left first molars. Laser parameters for each point were 660 nm, 35mW, 74.2 J/cm^2^, 2.10 J, spot 0.0283 cm^2^, contact, continuous mode.

### Tissue processing

The left hemimandibles were fixed in buffered 4% formaldehyde for 48 hr and demineralized in 10% ethylenediaminetetraacetic acid (EDTA) solution. Then, were submitted to conventional histological processing and paraffin embedding. Semi-serial 4 μm thick sections were obtained from buccal to lingual progression. Five equidistant sections from each specimen were stained with haematoxylin and eosin (H&E) for histopathological and histometric analyses [7]. Four additional sections from each specimen were subjected to the indirect immunoperoxidase method, using the following primary anti‐bodies: goat anti-osteocalcin (OCN) (Santa Cruz Biotechnology®) and goat anti-tartrate-acid phosphatase (TRAP) (Santa Cruz Biotechnology®). The indirect immunoperoxidase technique was conducted in accordance with Matheus et al. (2018) [25].

### Microscopy analysis procedure

Analyses were performed by a calibrated and masked staff using image analysis software (AxioVision 4.8.2; Carl Zeiss MicroImaging GmbH, Jena, TH, Germany). Histopathological analysis was conducted by a certified histologist [EE] based on the criteria described by Gusman et al. (2019) [26]. Each parameter was scored from 0 to 3 and is shown in Table 1. For the histometric analysis, the entire furcation (FA) was delineated and considered 100% of the area to be analyzed. The entire bone area (BA) was delineated within the limits of FA. The ratio of BA to FA was calculated and the results were expressed as the percentage of bone in the furcation (PBF). Remeasurement of the 5 sections stained with H&E was performed 1 week after the first measurement in order to assess the intra-examiner reliability and reproducibility by Kappa statistic.

**Table 1:** Scores and specimens’ distribution according to the parameters of the histologic analysis in groups NT, SRP, aPDT-pH7 and aPDT-pH1, with intragroup and intergroup comparisons.

HISTOLOGICAL ANALYSIS																		
SCORES FOR EACH PARAMETER	% ANIMALS PER SCORE																	
	EXPERIMENTAL GROUPS/TIMES																	
	NT				SRP				aPDT-pH7				aPDT-pH1					
	14d	22d	37d		14d		22d	37d	14d	22d	37d		14d		22d		37d	
**INTENSITY OF LOCAL INFLAMMATORY RESPONSE**																		
absence of inflammation	-	-	-		-		-	-	-	-	80		-		-		60	
small amount of inflammatory cells	-	-	-		-		40	100	-	100	20		-		100		40	
moderate amount of inflammatory cells	-	40	40		20		60	-	100	-	-		100		-		-	
large amount of inflammatory cells	100	60	60		80		-	-	-	-	-		-		-		-	
**MEDIAN**	**3**	**3**	**3**		**3**†		2*‡	1*‡	**2**†‡§	1*‡§	0*‡§		**2**†‡§		1*‡§		0*‡§	
**EXTENSION OF THE INFLAMMATORY PROCESS**																		
**0)** absence of inflammation	-	-	-		-		-	-	-	-	80		-		-		60	
**1)** extending to part of the connective tissue	-	-	-		-		40	60	100	100	20		100		100		40	
**2)** extending to the whole connective tissue	60	60	80		100		60	40	-	-	-		-		-		-	
**3)** extending to the whole connective tissue and to the alveolar bone	40	40	20		-		-	-	-	-	-		-		-		-	
**MEDIAN**	**2**	**2**	**2**		**2**		2*‡	**1**‡	**1**‡§	1*‡§	0*‡§		**1**‡§		1*‡§		1*‡§	
**EXTERNAL ROOT RESORPTION (CEMENTUM AND DENTIN)**																		
**0)** absence of external root resorption	-	-	-	40		20		-	40	-	-		40		-		-	
**1)** only inactive resorption areas	-	-	20	-		20		40	-	20	60		-		40		40	
**2)** few active resorption areas	60	60	60	60		60		60	60	80	40		60		60		60	
**3)** many active resorption areas	40	40	20	-		-		-	-	-	-		-		-		-	
**MEDIAN**	**2**	**2**	**2**	**2**‡		**2**‡		**2**	**2**‡	**2**‡	**1**		**2**‡		**2**‡		**2**	
**ALVEOLAR BONE RESORPTION**																		
**0)** within normality patterns	-	-	-	-		-		-	-	20	20	-		-		-		
**1)** small amount of alveolar bone resorption	-	-	-	-		20		40	20	40	60	40		40		80		
**2)** moderate amount of alveolar bone resorption	20	100	100	60		60		60	80	40	20	60		60		20		
**3)** large amount of alveolar bone resorption	80	-	-	40		20		-	-	-	-	-		-		-		
**MEDIAN**	**3**	**2**	**2**	**2**		**2** *		**2**	**2**‡§	1*‡§	**1**‡	**2**‡§		**2**		**1**‡		
**PATTERN OF THE CONNECTIVE TISSUE STRUCTURE**																		
**0)** moderate amount of fibroblasts and large amount of collagen fibers (dense connective tissue)	-	-	-	-			-	-	-	80	80	-		40		60		
**1)** moderate amount of fibroblasts and collagen fibers	-	-	-	-			60	60	-	20	20	-		60		40		
**2)** small amount of fibroblasts and collagen fibers	80	80	80	100			40	40	100	-	-	100		-		-		
**3)** severe tissue breakdown and areas with necrosis	20	20	20															
**MEDIAN**	**2**	**2**	**2**	**2**			1*‡	1*‡	**2**	0*‡§	0*‡§	**2**		1*‡§		0*‡§		
**PATTERN OF THE ALVEOLAR BONE STRUCTURE**																		
**0)** bone trabeculae with regular contour, surrounded by many active osteoblasts, including areas of new bone formation	-	-	-		-		-	-	-	-	20	-		20		40		
**1)** bone trabeculae with irregular contour, surrounded by many active osteoblasts and osteoclasts	20	20	20		20		100	100	20	100	80	20		80		60		
**2)** bone trabeculae with irregular contour, surrounded by active osteoclasts	80	80	80		80		-	-	80	-	-	80		-		-		
**3)** areas of necrotic bone and bone trabeculae with irregular contour, surrounded by many active osteoclasts	-	-	-		-		-	-	-	-	-	-		-		-		
**MEDIAN**	**2**	**2**	**2**		**2**		1*‡	1*‡	**2**	1*‡	1*‡	**2**		1*‡		1*‡		

Intragroup comparisons

*, statistically significant difference with 14 days at the same group (p≤0.05), †, statistically significant difference with 22 and 37 days at the same group (p≤0.05). Intergroup comparisons: ‡, statistically significant difference with NT at the same time points (p≤0.05); §, statistically significant difference with SRP at the same time points (p≤0.05). Statistical tests: Kruskal-Wallis and Dunn.

TRAP immunolabeling was evaluated by counting TRAP-positive cells at × 200 magnification within TA. A semiquantitative analysis of the immunolabelling of OCN was performed at × 400 magnification [26]. The immunolabelling was characterized as follows:

- Score 0: no immunolabelling (total absence of immunoreactive [IR] cells);
- Score 1: low immunolabelling (IR in ∼1/4 of cells per area);
- Score 2: moderate immunolabelling (IR in ∼1/2 of cells per area);
- Score 3: high immunolabelling (IR in ∼3/4 of cells per area).

### Primary and secondary outcomes

The primary outcome was defined as the bone formation within the furcation. Hence, PBF was considered the primary outcome variable. The secondary outcome was to define the tissues’ response and safety of the novel MB when compared with MB pH 7.0, through histopathological and immunohistochemical analyses.

### Statistical analysis

Data were analyzed using BioStat software (BioStat version 5.0, Belém, PA, Brazil). Cohen’s kappa coefficient was used to calculate the agreement between the measurements of PBF. The normality of distribution of the collect data was accessed by Shapiro‒Wilk test. The scores evaluated in the histopathological and immunohistochemical analysis of OCN were submitted to analysis of variance with Kruskal–Wallis test and post-test of multiple comparisons of Dunn (p≤0.05). Parametric data (PBF and TRAP) were analyzed with analysis of variance (One-way ANOVA) and post-test of multiple comparisons of Tukey (p≤0.05).

## Results

### Histometric analysis of PBF

**Fig 2:**
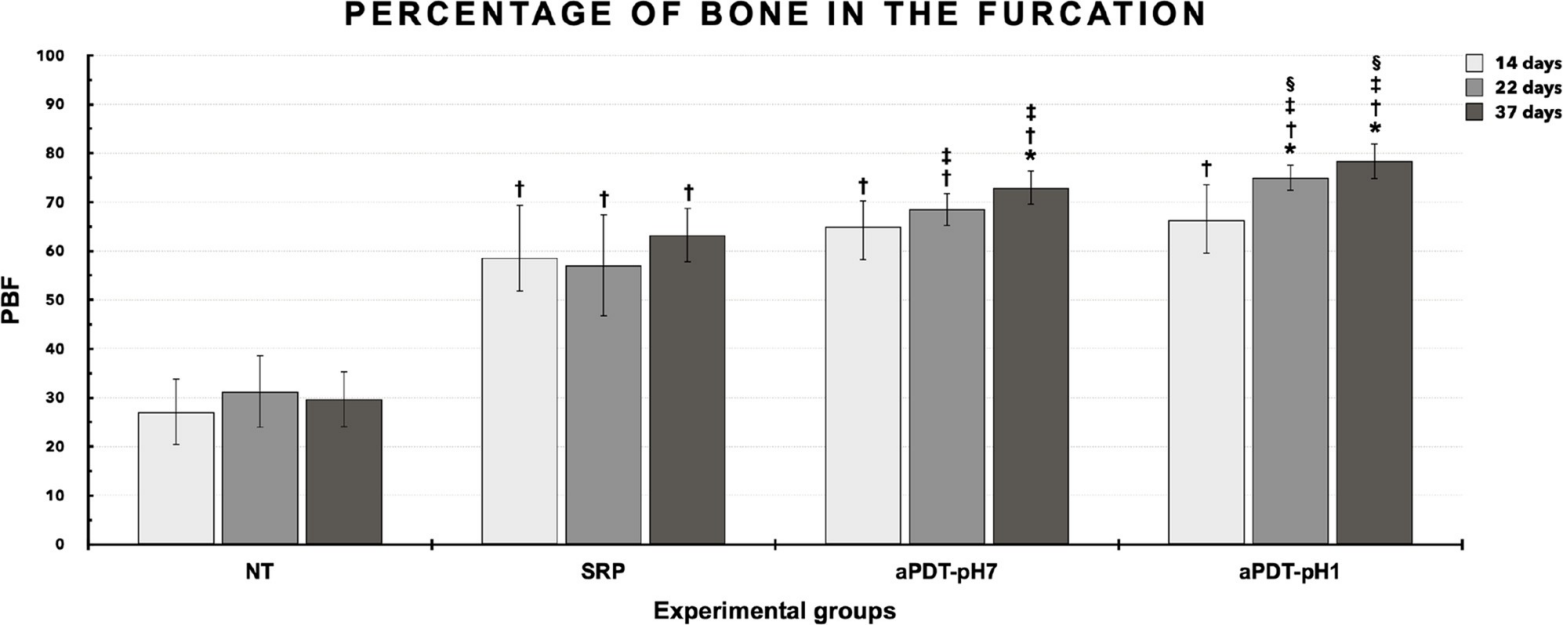
Graph showing the quantification (M±SD) of the percentage of bone in the furcation (PBF) of the mandibular left first molars for each group and period. Statistical tests: ANOVA and Tukey. Symbols: *, statistically significant difference with 14 days at the same group (p≤0.05); †, statistically significant difference with NT at the same time points (p≤0.05); ‡, statistically significant difference with SRP at the same time points (p≤0.05); §, statistically significant difference with aPDT-pH7 at the same time points (p≤0.05).

Cohen’s Kappa coefficient showed 96% agreement between measurements. The results of PBF are shown in Fig 2. In intragroup analysis, aPDT-pH7 showed lower PBF at 14 days (64.97%±5.38) compared with 37 days (72.96%±3.46) (p≤0.05). aPDT-pH1 showed lower PBF at 14 days (66.33%±7.35) compared with 22 (75.05%±2.66) and 37 days (78.4%±3.65) (p≤0.05). In intergroup analysis, group NT showed lower PBF at 14, 22 and 37 days (27.09%±6.85; 31.33%±7.41; 29.67%±5.68) when compared with groups SRP, aPDT-pH7 and aPDT-pH1 at the same time points (p≤0.05). Group SRP showed lower PBF at 22 (57.03%±10.46) and 37 days (63.24%±5.58) when compared with aPDT-pH7 and aPDT-pH1 at the same time points (p≤0.05). aPDT-pH7 showed lower PBF at 22 (68.48%±3.32) and 37 days (72.96%±3.46) when compared with aPDT-pH1 at the same time points (p≤0.05).

### Histopathological analysis of periodontal tissues

**Fig 3:**
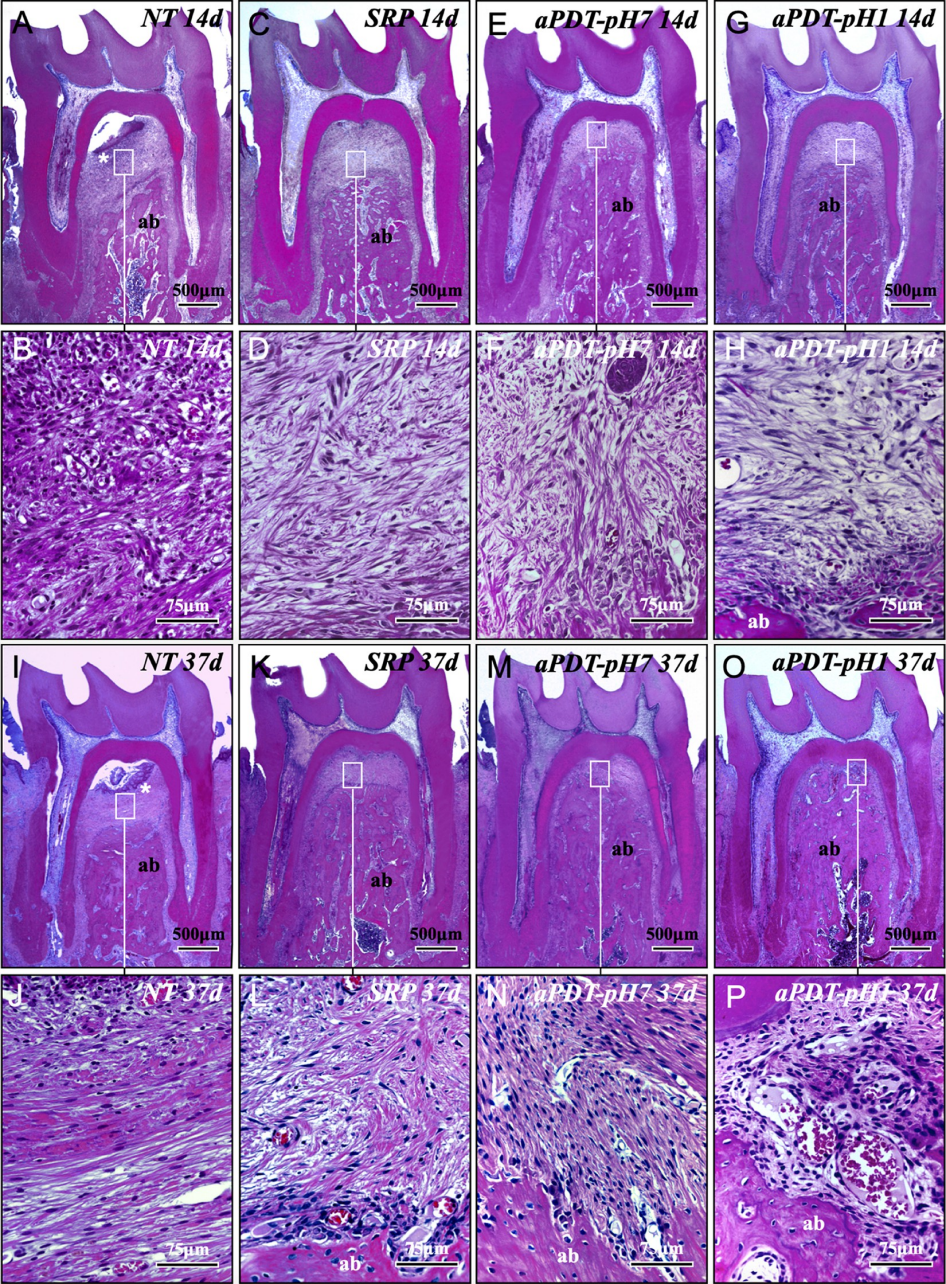
Histopathologic features of the periodontal tissues in the furcation region of the mandibular left first molars. Abbreviations and symbols: ab, alveolar bone; *, inflammatory infiltrate. a- h: photomicrographs showing the features of the periodontal tissues at 14 in NT (A, B), SRP (C, D), aPDT-pH7 (E, F) and aPDT-pH1 (G, H). I—P: photomicrographs showing the features of the periodontal tissues at 37 in NT (I, J), SRP (K, L), aPDT-pH7 (M, N) and aPDT-pH1 (O, P). A, C, E, G, I, K, M, and O allow an overview of the furcation, while B, D, F, H, J, L, N, and P provide a closer view of the inflammatory infiltrate, fibroblasts, arrangement of collagens fibers and alveolar bone in the furcation. Staining: Haematoxylin & Eosin. Scale Bars: A, C, E, G, I, K, M, and O: 500 μm; B, D, F, H, J, L, N, and P: 75 μm.

Experimental periodontitis severe in group NT, as confirmed by the higher extent of the local inflammatory response and progressive damage to periodontium when compared with the other groups. In group SRP was noticed decreased extent of local inflammatory response, as well as more favorable repair process over the experimental periods when compared with group NT. Groups aPDT-pH7 and aPDT-pH1 showed very similar histopathologic features, and when compared with group SRP, both presented reduced extent of local inflammatory response and greater tissue repair process after treatment (Fig 3). The parameters, scores, specimens’ distributions, and statistical analysis of periodontal tissues in NT, SRP, aPDT-pH7 and aPDT-pH1 are shown in Table 1.

### Immunohistochemical analysis

**Fig 4:**
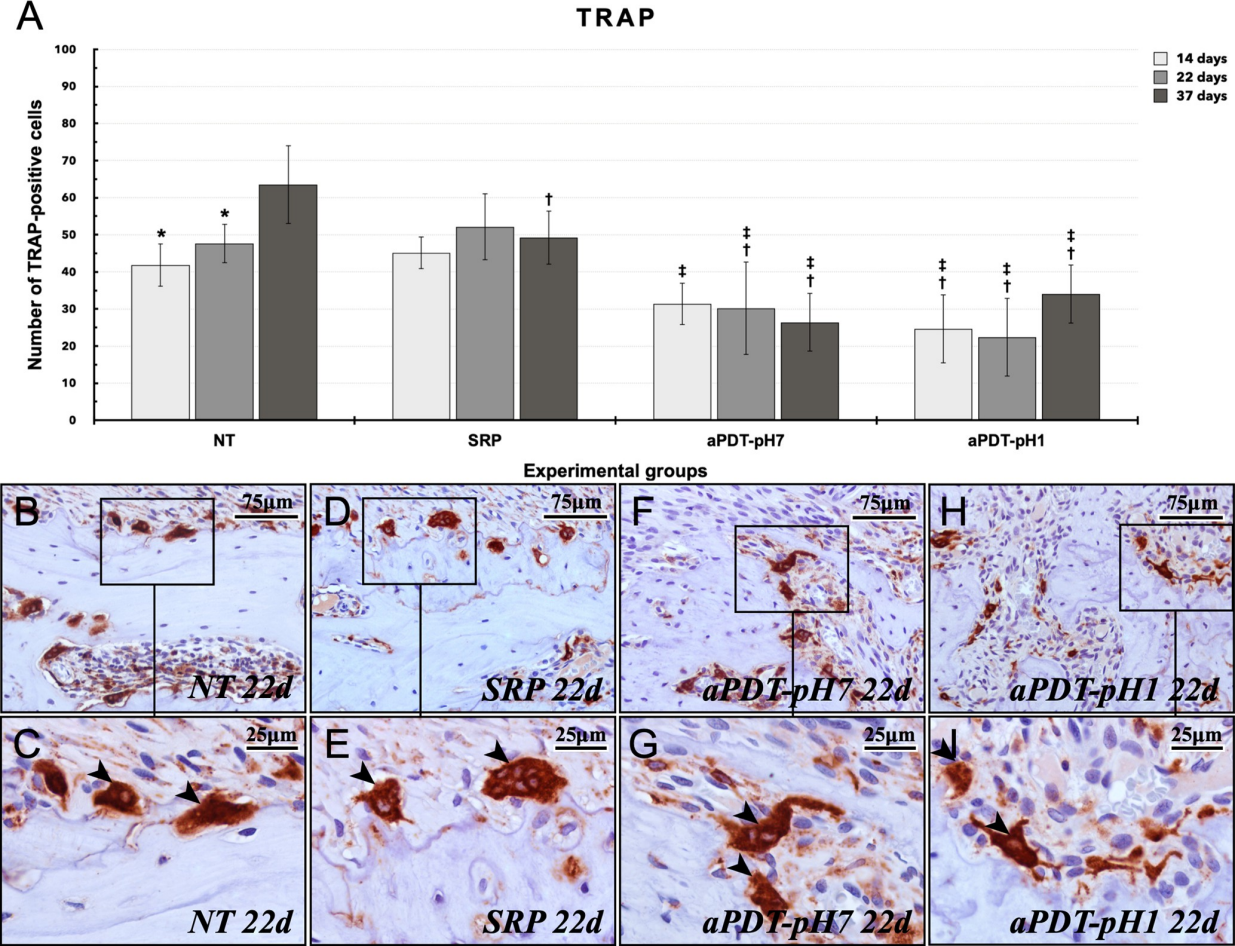
TRAP immunobelling in the furcation region of the mandibular left first molars at 22 days. (A) Means and standard deviations (M±SD) of the number of TRAP-positive cells in the furcation for each group and period. Statistical tests: ANOVA and Tukey. Symbols: *, statistically significant difference with 37 days at the same group (p≤0.05); †, statistically significant difference with NT at the same time points (p≤0.05); ‡, statistically significant difference with SRP at the same time points (p≤0.05). (B—I) Photomicrographs showing the TRAP-positive cells (black arrowheads) at 22 days in groups NT (B, C), SRP (D, E), aPDT-pH7 (F, G) and aPDT-pH1 (H, I). Counter-staining: Harris’ haematoxylin. Scale bars: B, D, F, and H: 75μm; C, E, G, and I: 25 μm.

The immunoperoxidase technique for detection of TRAP and OCN showed high specificity for these antigens, as confirmed by the absence of immunolabeling in negative control. In intragroup analysis, group NT showed higher number of TRAP-positive cells at 37 (63.5±10.5 cells) days when compared with 14 (41.8±5.80 cells) and 22 days (47.7±5.3 cells) (p≤0.05). In intergroup analysis, group NT (41.8±5.80; 47.7±5.3; 63.5±10.5 cells) showed higher number of TRAP-positive cells at 37 days when compared with group SRP (49.2±7.3 cells), at 22 and 37 days when compared with aPDT-pH7 (30.2±12.6; 26.4±7.9 cells), and in all experimental periods when compared with aPDT-pH1 (24.6±9.2; 22.4±10.6; 34±7.9 cells) (p≤0.05). Group SRP (45.2±4.4; 52.2±9; 49.2±7.3 cells) showed higher number of TRAP-positive cells at 14, 22 and 37 days when compared with aPDT-pH7 and aPDT-pH1 at the same time points (p≤0.05) (Fig 4).

**Fig 5:**
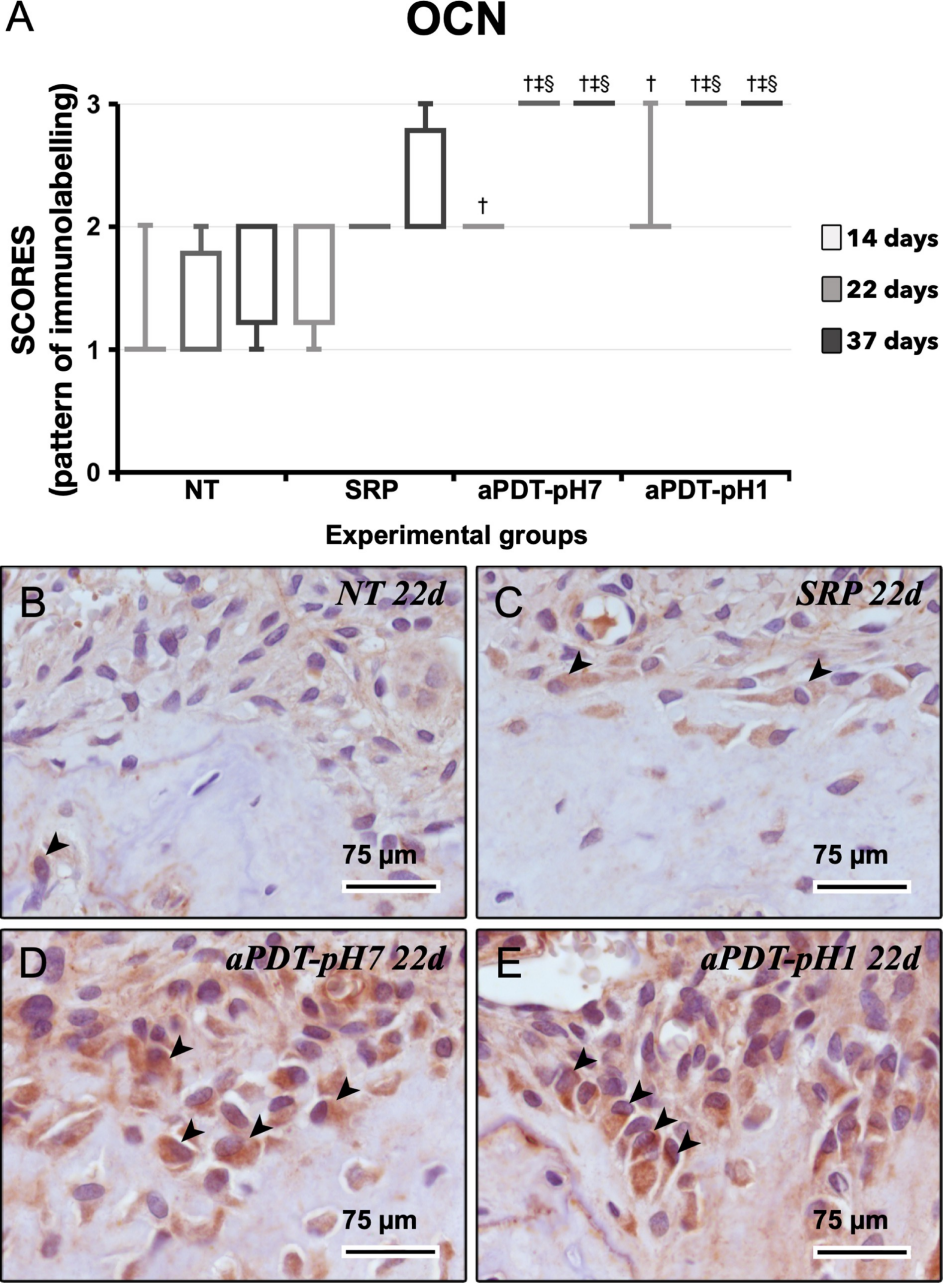
OCN immunobelling in the furcation region of the mandibular left first molars at 22 days. (A) Graph showing the median and interquartile range of the scores for OCN in the furcation for each group and period. Statistical tests: Kuskal-Wallis and Dunn. Symbols: ‡, statistically significant difference with 14 days at the same group (p≤0.05); †, statistically significant difference with NT at the same time points (p≤0.05); §, statistically significant difference with SRP at the same time points (p≤0.05). B–E: photomicrographs showing the immunolabelling pattern for OCN and OCN-positive cells (black arrowheads) at 22 days in groups NT (B), SRP (C), aPDT-pH7 (D) and aPDT-pH1 (E). Counter-staining: Harris’ haematoxylin. Scale bars: 75 μm.

In intragroup analysis for OCN, aPDT-pH7 and aPDT-pH1 showed lower immunolabeling pattern at 14 days when compared with 22 and 37 days (p≤0.05). In intergroup analysis, group NT showed no statistically significant difference with group SRP and lower immunolabeling pattern when compared with groups aPDT-pH7 and aPDT-pH1 in all experimental periods (p≤0.05). Group SRP showed lower immunolabeling pattern when compared with groups aPDT-pH7 and aPDT-pH1 at 22 and 37 days (p≤0.05) (Fig 5).

## Discussion

To target more than one system and/or tissue enrolled with periodontal treatment and repair (i.e. microbiota, specific microenvironment, host immune and inflammatory systems, bone, root surface, and collagen fibers) may be the key for adjunctive therapies to provide standout additional benefits over traditional forms of treating periodontitis. The present study evaluated the effectiveness and safety of a citric acid-based MB pH 1.0 to be used with aPDT as adjunctive therapy to SRP for the treatment of experimental periodontitis. The results support that the acidic MB is as safe as the conventional MB (pH 7.0) used with aPDT so far (i.e. similar histopathologic features, tissues’ reaction, and expression of bone-related biomarkers). Additionally, our hypothesis that the acidic MB pH 1.0 could provide additional benefits over conventional MB was confirmed by the higher PBF in group aPDT-pH1 at 22 and 37 days, when compared with aPDT-pH7.

The methodology of EP induction by ligature is an experimental model widely used in researches evaluating periodontal diseases [7, 22]. The histopathologic features obtained 7 days after ligature placement are very similar [22] to those observed in humans, and, also, the equivalent complexity of the microbiota is confirmed by the absence of substantial periodontal breakdown in *“germ-free”* animals [27]. Furthermore, animal studies might mimic the closest situation to the clinical scenario in order to establish valuable cause and effect relationships. On this topic, the reduced dimensions and the impaired access to the furcation of rats’ molars validate the application of adjunctive therapies for the treatment of EP in these areas [6]. Also, neither maintaining (present experiment) nor removing the ligature 7 days after induction would compromise the comparisons in this model, since the removal of the ligature didn’t prevent the occurrence of alveolar bone loss in the furcation [28]. Hence, this experimental model is capable to validate the hypothesis and to confirm the security and effectiveness of novel therapies for the treatment of periodontal disease in humans [22].

The innate host molecules and inflammatory cells are significantly different when healthy and diseased sites are compared. Although bacteria are present in healthy sites, they coexist in a homeostatic relationship with periodontal tissues, in which an orchestrated expression of select innate host-defense mediators are observed [29, 30]. Conversely, periodontitis sites disorderly express more and different mediators compared with healthy sites [3, 31]. aPDT is known to target microorganisms related to the pathogenesis of periodontitis, however, it was hypothesized by Braham et al. [32] that aPDT may promote periodontal healing not only by killing bacteria but also by inhibiting destructive host responses. Hence, the higher biocompatibility of MB pH 1.0 found by Gusman et al. [33] is likely to support some of the better results found in group aPDT-pH1.

Persistence on the expression of prostaglandin-E_2_ harms bone formation through upregulation of RANKL and inhibition of osteoprotegerin [34]. Osteoblasts and osteoclast are two of the main cells responsible for bone metabolism and maintenance of the hierarchical structure of bone [35]. Osteocalcin regulates bone mineralization and turnover [36], while TRAP is expressed from differentiation until apoptosis of osteoclasts [37], therefore, both proteins are important regulators of the bone tissue. With regard to the expression of both biomarkers, this experiment showed that both aPDT protocols (aPDT-pH7 and aPDT-pH1) reduced the number of TRAP-positive cells and increased the immunolabelling pattern of OCN in the furcation when compared with only SRP.

Noteworthy, there is a strict relationship between alveolar bone loss and the success of periodontal therapies [38]. In spite of a possible positive effect of aPDT with MB acidulated with citric acid at pH 1.0 for the treatment of periodontitis, as evidenced by the increased PBF at 22 and 37 days, when compared with 14 days in group aPDT-pH1, further experimental research pursuing deep knowledge on this topic, as well as researches evaluating other parameters shall be conducted in order to eventually translated their results to the clinical scenario.

Even though the mechanisms by which the acidic MB increased the amount of alveolar bone when compared to neutral MB are not completely elucidated, it is known that the interaction of citric acid with mineralized surfaces leads to ionic-exchange processes, increasing the release of Ca^++^ [39] and precipitation of Ca_3_(citrate)_2_.4H_2_0 onto apatite surface [40], plausible triggers for bone formation. Furthermore, this beneficial property over molecules that positively affect bone might not be understood as limited to bone tissue, since calcium and calcium citrate regulate fundamental cellular events, such as mitochondrial activity [41]. Also, despite the distinct purpose and methods from this experiment, de Rezende et al. [42] observed that demineralization of bone surfaces with citric acid pH 1.0 provided an appropriate substrate for pre-osteoblast proliferation and bone mineralization.

Innumerous PS have been used with aPDT for the treatment of periodontitis [43]. Curcumin [44], toluidine blue [45], chlorin [46], and MB [47] are able to promote a significant antifungal and antibacterial effect against microorganisms. However, the impaired penetration of PS deep in the biofilm matrix decreases the effectiveness of the few PS molecules that are able to penetrate the biofilm matrix [48]. Such drawbacks can be overcome by the development of modified PS [49]. Most experiments confirmed the efficacy of the optimization of MB derivatives to kill bacteria [50, 51] but usually fail to assess their effects on periodontal tissues.

Negatively charged citric acid is believed to inactivate bacteria by either destabilizing the outer membrane or by sequestering essential metals from the growth environment [52, 53]. Recently, Burel et al. [54] evaluated the impact of pH on citric acid antimicrobial activity against Gram-negative bacteria. Their [54] results indicate that tribasic citric acid was the most effective in damaging the bacterial membrane. However, a reduction in the absolute number of bacteria and damages to cell walls were found regardless of the tested pH. No similar experiment has been carried in order to evaluate the effectiveness of citric acid on targeting the membrane or killing periodontal pathogens. Hence, the promising findings of Burel et al. [54] encourage further research on the plausible efficacy of using citric acid-based therapies for reducing bacterial colonization related to the pathogenesis of periodontitis.

The harm to tissues is reasoned by the term “acid strength”, described as the potential of acid to lose protons H+. For instance, this property can be adjusted by topology structure and morphology, and crystallinity in addition to the chemical composition [55]. Although in a contaminated environment no statistical significance in inflammatory parameters has been observed between acidic and neutral MB, a subcutaneous test showed the best biocompatibility for MB pH 1.0 [22] as compared with MB pH 7.0.

The use of citric acid for decontamination and repair has being the focus of *in vitro* and *in vivo* experiments in implantology. Souza et al. [19] demonstrated that citric acid significantly reduced the biofilm related with peri-implantitis, as well as enhanced the electrochemical stability of titanium. Htet et al. [56] observed increased bone formation following the combination of mechanical and chemical treatment with citric acid for disinfection of anodized implant surface, which may encourage further research using the acidic MB for the treatment of peri-implantitis as well.

To the best of our knowledge, this is the first experiment to investigate the influence of an acidic dye used in aPDT for the treatment of EP in an *in vivo* model. As with any animal model, the data presented by this experiment shall be interpreted with caution, since the contact of acidic substances with denuded root surface is frequently related with dentin hypersensitivity in humans.

Collectively, our data indicate that aPDT using the citric acid-based MB pH 1.0 is as secure for periodontal tissues as the conventional MB (pH 7.0) used in aPDT so far. Our findings suggest an additional benefit of stimulating bone formation in the furcation following aPDT with the acidic MB.

## Supporting information

S1 Dataset POF dataset. (PDF)

S2 Dataset TRAP dataset. (PDF)
