# Supplementary material for: Evaluation of antimicrobial photodynamic therapy with acidic methylene blue for the treatment of experimental periodontitis
Source: PLoS One. 2022 Feb 10;17(2):e0263103. doi: 10.1371/journal.pone.0263103 (PMC8830666; doi:10.1371/journal.pone.0263103)
Supplement: S1 Dataset — (PDF) [file pone.0263103.s001.pdf]

POF dataset

	GROUPS											
**ANIMALS**	**NT 14 days**	**NT 22 days**	**NT 37 days**	**SRP 14 days**	**SRP 22 days**	**SRP 37 days**	**aPDT-pH7 14 days**	**aPDT-pH7 22 days**	**aPDT-pH7 37 days**	**aPDT-pH1 14 days**	**aPDT-pH1 22 days**	**aPDT-pH1 37 days**
**R1**	36,96	36,42	39,89	67,43	67,32	65,69	67,98	68,3	75,6	68	78,1	84,3
**R2**	36,89	36,65	39,69	67,31	39,75	66,01	66,49	70,5	75,66	59,2	80,02	79,58
**R3**	32,97	34,6	23,99	43,69	56,67	65,43	64,78	72,81	69,35	73,65	73,21	83,66
**R4**	19,34	24,76	31,45	56,76	67,3	49,28	69,68	72,25	68,4	74,3	73.78	77,38
**R5**	21,94	41,84	28,45	67,3	69,1	62,46	66,56	70,25	68,74	56,43	72,45	77,54
**R6**	27,99	40,8	26,69	67,98	65,31	65,30	66,93	62,95	75,34	53,56	74,39	79,76
**R7**	29,94	24,99	25,3	54,35	54,67	65,12	65,67	64,25	69,34	69,43	73,56	77,46
**R8**	23,39	23,56	26,78	59,65	42,78	68,00	69,78	70,23	75,45	68,12	77,54	71,98
**R9**	22,15	23,91	27,65	43.76	49,87	58,38	59,89	67,23	75,8	72,45	73,56	76,34
**R10**	19,28	25,8	26,85	42,98	57,48	66,68	51,89	66	75,89	68,2	72,56	75,95
**MEANS**	27,09	31,33	29,67	58,61	57,03	63,24	64,97	68,48	72,96	66,33	75,04	78,40
**SD**	6,85	7,41	5,68	10,04	10,46	5,58	5,38	3,32	3,46	7,35	2,77	3,65
