# Supplementary material for: Evaluation of antimicrobial photodynamic therapy with acidic methylene blue for the treatment of experimental periodontitis
Source: PLoS One. 2022 Feb 10;17(2):e0263103. doi: 10.1371/journal.pone.0263103 (PMC8830666; doi:10.1371/journal.pone.0263103)
Supplement: S2 Dataset — (PDF) [file pone.0263103.s002.pdf]

TRAP dataset

	GROUPS											
ANIMALS	NT 14 days	NT 22 days	NT 37 days	SRP 14 days	SRP 22 days	SRP 37 days	aPDT-pH7 14 days	aPDT-pH7 22 days	aPDT-pH7 37 days	aPDT-pH1 14 days	aPDT-pH1 22 days	aPDT-pH1 37 days
R1	41	49	53	44	37	53	44	45	22	12	31	43
R2	39	49	73	46	51	45	44	41	32	21	30	42
R3	44	48	74	39	53	63	29	42	22	37	29	31
R4	34	39	56	55	40	43	28	14	35	17	38	25
R5	36	53	57	45	52	54	27	28	34	37	23	26
R6	38	49	71	45	51	44	29	42	37	24	13	39
R7	40	37	78	47	50	45	28	37	29	14	30	38
R8	54	52	52	39	65	39	28	20	15	23	10	44
R9	45	51	70	46	60	50	28	19	20	35	10	26
R10	47	50	51	46	63	56	29	14	18	26	10	26
MEANS	41,80	47,70	63,50	45,20	52,20	49,20	31,40	30,20	26,40	24,60	22,40	34,00
SD	5,884	5,355	10,575	4,467	9,004	7,300	6,670	12,559	7,905	9,204	10,679	7,944
